# Is Pet Ownership Helpful in Reducing the Risk and Severity of Geriatric Depression?

**DOI:** 10.3390/geriatrics1040024

**Published:** 2016-10-04

**Authors:** Nancy J. Needell, Nisha Mehta-Naik

**Affiliations:** Department of Psychiatry, Weill Cornell Medical College, 525 E. 68th Street, Box 140, New York, NY 10065, USA; nim9063@nyp.org

**Keywords:** pet ownership, animal assisted therapy, older adults, depression, loneliness

## Abstract

Many community-dwelling older adults are searching for ways to remain mentally and physically healthy as they age. One frequently offered suggestion is for older people to adopt a pet to avoid loneliness, to stay socially engaged, and to stave off depression. Despite the ubiquity of this advice in popular culture, research findings are equivocal on whether pet ownership is beneficial to the physical and psychological health of older adults. This article evaluates published data relating to pet ownership and its possible impact on depression and related symptoms in the elderly.

## 1. Introduction

Depression among the elderly has serious medical, social, and financial sequelae for those who suffer from it as well as for their caregivers. Multiple studies have documented the medical co-morbidities, decreased life expectancies, and other physical complications that accompany untreated and incompletely treated depression in the elderly [1]. The indirect financial costs to society attributable to geriatric depression—for example, the lost economic productivity and the personal suffering associated with caregiving, or the costs of residential treatment—are just beginning to be appreciated [2].

As a result, many older people seek ways to avoid depression or to use psychosocial adjuncts to augment medication and traditional psychotherapies. Senior centers, adult education classes, and group exercise classes, among other community-based programs, are social programs designed to keep older people engaged and less isolated with the hope that increased socialization will lead to improved mental health.

Another idea that has gained traction recently is pet ownership. Pets, particularly dogs and cats, are seen not only as providers of unconditional love for their elderly owners but also as companions and a means through which otherwise isolated seniors may re-establish human connections. A pet also helps an otherwise sedentary senior remain active, which in itself may confer physical and psychological benefits. Popular media is replete with the images of older adults and their “four-legged friends” [3,4].

However, pet ownership may not necessarily be beneficial for an older person, especially one with a mental or physical disability. Pets can be costly and can consume much time and energy. They can be noisy and disruptive to neighbors and can place elderly owners at risk of falls at home or while on walks. There is also the question of how an older person, already facing multiple losses, will deal with the death of a beloved pet or with choosing between caring for oneself or for one’s pet.

The lead author, the Medical Director of a geriatric-focused Psychiatric Mobile Crisis Team, has seen multiple older patients with mental and physical health issues for whom pet ownership presented a barrier to treatment. In one case, an elderly patient refused necessary in-patient psychiatric admission because she had no one to care for her beloved cat. In another, near-tragic situation, a severely depressed man in his 80s did not leave the house for almost two months, feeding himself and his dog only what was in the home; when the Mobile Crisis Team arrived, they were subsisting on salad dressing and mayonnaise. A third patient, a retired older nurse, hid her frailty and loss of function out of fear that she would be separated from her dog if placed in a nursing home. Mobile Crisis was called after she had not seen a doctor or refilled prescriptions in months, requiring a medical hospitalization.

## 2. Methods

For this article a literature review was conducted to assess the current state of knowledge on pet ownership and depression in older adults. Each author conducted an independent literature review. The PubMed/MedLine database was accessed using the MeSH terms “older adults”, “elderly”, “depression”, “loneliness”, “pets”, “pet ownership”, and “animal-assisted therapy”. Only articles in English published after 1990 were included. Studies focused on people whose primary diagnosis was dementia or other cognitive impairment were excluded. The reference sections of published articles were reviewed for citations to relevant works. Given the relatively sparse literature available, multiple types of publications were reviewed, including empirical research, position papers, editorials, and case reports. Journals included medical and psychiatric publications, anthropologic and sociology publications, and veterinary publications. Studies of “animal-assisted therapy” were included as this material had relevant research findings related to depression and animal involvement in older adults.

A total of 21 scholarly articles were ultimately included. They were evaluated for relevance to the prevention or treatment of geriatric depression, methodology, subject selection, and findings. Some studies addressed geriatric depression directly while others focused on related conditions, such as loneliness, social isolation/connectness, perceptions of overall health and well-being, and interactions with the medical community.

## 3. Results

The most consistent finding throughout all studies was the lack of definitive conclusions; no study showed that pet ownership conferred either clear benefits or clear risks regarding the mental health of elderly people.

### 3.1. What Is the Relationship between Pet Ownership and Depression in the Elderly?

Five studies directly addressed the question of whether pet ownership was correlated with lower levels of depression in older adults. None found overwhelming evidence to support the popular assertion that owning a pet contributes to the alleviation of depression. Details are shown in Table 1.

#### 3.1.1. Positive Effect on Depression

One telephone survey of 201 adults ranging in age from 19 to 94 years old found decreased levels of depression in unmarried, dog owners. Levels of depression in married dog owners were not affected. The survey also found that dog ownership was associated with a greater sense of self-reported personal life satisfaction (though a lower sense of wellbeing for men) [5]. A study of post-myocardial infarction patients found that depressed post-MI patients who owned pets had better medical outcomes and lower mortality rates than depressed post-MI patients who did not own pets. However, pet ownership itself did not decrease primary rates of depression; thus pet ownership in that study appeared to moderate the effects, if not the rates, of depression [6].

#### 3.1.2. Absent or Exacerbating Effect on Depression

The survey of 201 adults found that dog ownership had no effect on depression overall but that dog ownership was associated with lower levels of depression in younger, but not older, adults [5]. Similarly, the study of post-myocardial infarction patients found that pet ownership did not decrease overall rates of depression [6]. A survey of older pet owners in Australia found that pet owners did not report better mental or physical health nor did they use fewer services. That study found a small, but not statistically significant, exacerbating effect of pet owners reporting higher scores on the Short-Form Health Survey (SF-12) than non-pet owners, indicating that some pet owners reported higher depression scores than their non-pet owning counterparts. The authors concluded that “on average, both pet owners and carers in this study reported significantly more depressive symptoms” than people without pets [7]. In a similar vein, a study of clients of two veterinary clinics and one pet grooming salon found that adults with high levels of attachment to their pets actually reported higher levels of depression than those with lower self-reported levels of attachment. That study also included the important observation that “elders who cannot adequately provide for their dog have higher levels of depression than those who can” [8]. An epidemiological study in Norway found that self-reported rates of depression were higher in cat owners than in dog owners or non-pet owners [9].

### 3.2. What Is the Relationship between Pet Ownership and Traits/Symptoms Associated with Depression in the Elderly?

Multiple studies and reviews looked at factors that can lead to or accompany depression in older adults to see what effect, if any, pet ownership had on their presence or severity. These factors can be relevant on their own or could be seen as “surrogates” for depression. Details are shown in Table 2.

#### 3.2.1. Loneliness and Pet Ownership

The most frequently studied “surrogate for depression” was loneliness. One study of 175 women in senior centers and pet-friendly senior living residences found that pet owners self-reported less loneliness than non-pet owners. [10]. The same lead author, in another analysis of the same data, concluded that pet attachment mediated the effect of loneliness in older women [11]. In a study of older patients in a primary care medical setting, researchers found that pet owners were 36% less likely than non-pet owners to report loneliness [12]. Confounding the issue, though, was a review of data from 5210 people over 50 in England that revealed a complex interaction between pet ownership and loneliness—“pet ownership can be a response to loneliness for the always lonely and a protection for those who recovered from loneliness.” Their study found that having a pet increased the likelihood of self-reported loneliness among females and that women who were lonely were more likely to own pets [13].

#### 3.2.2. Social Engagement and Pet Ownership

The social connectedness that can accompany pet ownership was also reviewed. One comprehensive literature review of all types of human–animal interactions concluded that “pets and companion animals seem to reduce psychological stress by altering the owner’s perceptions and making situations and people seem more benign”. The author noted, however, that the “impact of pet ownership seems to be most important for highly stressed or socially isolated individuals” [17]. Social isolation was at the heart of a more sociologically based study that found that pets, particularly dogs, “can serve as a kind of medium through which social contacts can be established in everyday situations”. Pets, the authors noted, can help older people structure their day and be a point of interaction with other people [14].

#### 3.2.3. Activities of Daily Living and Pet Ownership

Raina et al, in a telephone survey of community-dwelling people over 65, found that Activities of Daily Living (ADLs) declined further in non-pet owners than in pet owners. The study found no statistical significance in assessing whether pet ownership impacted psychological well-being in older adults [15].

#### 3.2.4. Somatic Issues and Pet Ownership

Pets can serve as a way for older adults to engage in society at large, but they can also be a way for isolated older adults to engage with the health care system and other agencies. One author opined that pet ownership might be a way to reach otherwise disenfranchised older Latinos, for whom animals are often more than companions or pets [18].

Other studies have looked at the impact of pet ownership on medical health, specifically at the number of medical visits made by older adults with pets versus those without pets. A study of one large group of one health care maintainence Medicare program recipients showed that people with pets had fewer patient-initiated doctor visits and concluded that “owning a dog provided a stress buffer, whereas other types of pets did not.” By “stress buffer”, the authors meant that dog owners were better able to tolerate normal stressful life events, specifically the loss of loved ones [16]. A large Norwegian study showed an increase in overall positive health–related characteristics in older pet-owning adults, other than finding that cat owners had higher body mass indexes (BMIs) than non-cat owners [9].

### 3.3. What Specific Risks Are Posed by Pet Ownership in the Elderly?

Fewer studies looked at the potential downside of pet ownership among the elderly. One author discussed many problems that older pet owners might face. Lifetime costs of pet ownership average between $8000 and $10,000 [19]. Pets can serve as vectors for disease, can contribute to falls, and can cause conflict with neighbors [19], and, of course, pets die. Older people often have older pets and there is no guarantee that the person will outlive the pet. Surprisingly, the literature search did not highlight studies that investigated the specific issue of grief and depression in older adults after the death of a pet, although it was discussed in at least two studies [15,20].

There were, however, two articles about attempted or completed suicide that included a beloved pet in the action. In one case, a woman with depression brought her dog with her into the garage, where she turned on the engine of her car, killing both herself and her dog [21]. Another author cited one case in which an older woman tried to strangle her dog before she took an overdose; the dog escaped and the woman survived and after hospitalization, she was reunited with her dog. The same author also reported on an elderly woman who gave her dog away before attempting suicide; after treatment, she too was reunited with her pet [22].

### 3.4. What Benefits Are Conferred by Association with Pets without the Full Commitment of Pet Ownership?

Animal-assisted therapy is increasingly used in working with the elderly; it is not uncommon to see animals visiting nursing homes, hospitals, and senior centers. This option allows older adults to have access to animals without assuming the financial, emotional, social, and physical obligations of pet ownership. While most studies of the psychological value of animal assisted therapy have focused on people with dementia and cognitive impairment, some address whether having access to animals has any impact on depression and overall psychological well being.

In a review of the literature, Cherniack and Cherniack found “mixed results” in the use of animals in people with depression and schizophrenia. They noted that several “trials of animal-assisted therapy demonstrated improvements in behavioral symptom scores in small numbers of subjects of limited duration” but stopped short of giving a full endorsement to this form of therapy [23]. Another large literature review looked at animal-assisted interventions for elderly patients with dementia and associated disorders. These authors noted that the wide disparity in study design and execution made it difficult to come to a consensus abut the efficacy of using animals in this population. They did note that animal involvement helped in “calming of agitated behavior and (had) positive effects on quality of social interaction and mood disturbances”, which could have relevance for depressed cognitively intact elders [24].

A small study of 16 elderly residents of a South African nursing home reviewed the Beck Depression and Anxiety Inventory (BDI and BAI) scores before and after six weeks of visits with a therapy pet. These were compared with controls who did not see the animal. Mean BDI scores went down from 19.86 to 11.86 (*p* = 0.017) in the animal group compared with the control group with no change noted in anxiety scores. The authors further noted that the people in the animal group reported mood and psychological benefits that were not captured by the ratings scales [25]. A study of elderly psychiatric inpatients with diagnoses of dementia, depression, and psychosis showed a 50% improvement on the Geriatric Depression Scale and a four-point improvement on the Mini-Mental State Examination in patients exposed to pet therapy compared with a control group [26].

## 4. Discussion

At present, there is insufficient evidence to back the claim in popular culture that pet ownership is helpful in preserving the mental health of older adults. Loneliness and social isolation seem to be ameliorated by pet ownership, but this does not necessarily correlate to lower rates of depression or reduction in the severity of depressive symptoms.

This review has several limitations. There was a relative paucity of research on the direct connection between pet ownership and geriatric depression and what studies were available had vastly differing methodologies and subject pools. In many cases, “markers” or “stand-ins” for depression, most notably, loneliness and social isolation, were studied rather than depression itself. This can be misleading, as not all people who are socially isolated or lonely meet criteria for major depression and many older people who do meet criteria for major depression are not lonely or isolated. There was no study directly addressing the percentages of older pet owners with depression versus the percentage of older non-pet owners with depression.

Future studies might look at whether older pet owners with depression have new onset of illness in later years or whether they have had depressive episodes over their lifetimes. Studies might also investigate whether lifelong pet owners have lower lifetime rates of geriatric depression than do those who have never owned pets. It would be valuable to investigate whether geriatric depression remits when a person becomes a pet owner as well as whether geriatric depression worsens when a person is less able to care for a pet or when that pet gets ill or dies.

Finally, a study could could compare pet owners with people involved in animal-assisted therapy to assess for rates of depression as well as rates of loneliness and other “markers”. It is possible that animal-assisted therapy and other means of connecting older people with animals without the entanglements and commitment of actual pet ownership is a preferable option in some cases.

Clinicians and other service providers need to be aware of the potential benefits of pet ownership but also of the needs that might arise in older adults caring for pets. Mental health providers might consider partnering with veterinary practices, pet stores and shelters, pet service agencies (such as groomers and dog walkers), and animal-assistance organizations to help their older patients get the most out of their relationships with their pets while reducing the burden of caring for them. Clinicians should be wary of suggesting that their older patients become pet owners as a way of maintaining mental and physical health. They should investigate whether their patients have the financial, physical, and psychological means to care for both themselves and a pet. As an alternative, animal-assisted therapy might allow them the connection with an animal while alleviating them of the burdens of pet ownership.

## Figures and Tables

**Table 1:** Studies of pet ownership and depression.

Study	Subjects	N	Findings
**Cline [5]**	Adults	201	Younger—but not older—dog owners had lower levels of depression than non-dog owners
**Friedmann et al. [6]**	Post myocardial infarction patients	460	Depressed pet owners had better medical outcomes than depressed non-dog owners
**Parslow et al. [7]**	Adults 60–64	2251	Pet owners reported higher levels of depressive symptoms than non-pet owners
**Miltiades et al. [8]**	Clients of veterinary clinics and dog groomers over 60	117	High self-reported attachment to pets correlated with higher self-reported depression
**Enmarker et al. [9]**	Adults 65–101	12,297	Cat owners had higher levels of depression than dog owners and non-pet owners

**Table 2:** Studies of pet ownership and symptoms associated with depression.

Study	Subjects	N	Findings
**Krause-Parello [10,11]**	Pet-owning women aged 55–84	159	Pet owners reported less loneliness than non-pet owners
**Stanley et al. [12]**	Patients in a primary care medical practice aged 60 and above	830	Pet owners are less likely to self report loneliness than non-pet owners
**Pikhartova et al. [13]**	Adults aged 50 and above	5210	Pet owners are more likely to self report loneliness; women who are lonely are more likely to own pets than women who are not lonely
**Scheibeck et al. [14]**	Unmarried dog owners aged 70 and above	23	Pet ownership helps older adults structure their day and can help facilitate social interaction
**Raina et al. [15]**	Adults aged 65 and above	995	Pet owners had less decline in Activities of Daily Living than non-pet owners
**Siegel [16]**	Adults aged 65 and above	1034	Pet owners initiate fewer visits to physicians than non pet owners
